# The effect of dietary red palm oil on the functional recovery of the ischaemic/reperfused isolated rat heart: the involvement of the PI3-Kinase signaling pathway

**DOI:** 10.1186/1476-511X-8-18

**Published:** 2009-05-29

**Authors:** Anna-Mart Engelbrecht, Louise Odendaal, Eugene F Du Toit, Kristina Kupai, Tamás Csont, Peter Ferdinandy, Jacques van Rooyen

**Affiliations:** 1 Department of Physiological Sciences, University of Stellenbosch, Stellenbosch, 7600, South Africa; 2 Department of Medical Physiology, University of Stellenbosch, Stellenbosch, 7600, South Africa; 3 Cardiovascular Research Group, Department of Biochemistry, University of Szeged and Pharmahungary™ Group, Szeged, Hungary; 4 Faculty of Health and Wellness Sciences, Cape Peninsula University of Technology, 7535, Bellville, South Africa

## Abstract

We have previously shown that dietary red palm oil (RPO) supplementation improves functional recovery in hearts subjected to ischaemia/reperfusion-induced injury. Unfortunately, the cellular and molecular mechanisms responsible for this phenomenon are still poorly understood and no knowledge exists regarding the effects of RPO supplementation on the phosphoinositide 3-kinase (PI3-K) signaling pathway and apoptosis during ischaemia/reperfusion injury. Therefore, the aims of the present study were three fold: (i) to establish the effect of RPO on the functional recovery of the heart after ischaemia/reperfuion injury; (ii) to determine the effect of the PI3-K pathway in RPO-induced protection with the aid of an inhibitor (wortmannin); and (iii) to evaluate apoptosis in our model. Wistar rats were fed a standard rat chow control diet or a control diet plus 7 g RPO/kg for six weeks. Hearts were excised and mounted on a Langendorff perfusion apparatus. Mechanical function was measured after a 25 min period of total global ischaemia followed by 30 minutes of reperfusion. Hearts subjected to the same conditions were freeze-clamped for biochemical analysis at 10 min during reperfusion to determine the involvement of the PI3-Kinase signaling pathway and apoptosis in our model. Dietary RPO supplementation significantly increased % rate pressure product recovery during reperfusion (71.0 ± 6.3% in control *vs *92.36 ± 4.489% in RPO; p < 0.05). The % rate pressure product recovery was significantly reduced when wortmannin was added during perfusion (92.36 ± 4.489% in the RPO group *vs *75.21 ± 5.26% in RPO + Wm). RPO + Wm also significantly attenuated PI3-K induction compared with the RPO group (59.2 ± 2.8 pixels in RPO vs 37.9 ± 3.4 pixels in RPO + Wm). We have also demonstrated that PI3-K inhibition induced PARP cleavage (marker of apoptosis) in the hearts during ischaemia/reperfusion injury and that RPO supplementation counteracted this effect.

## Background

Cardiovascular disease is one of the major causes of death in the Western world. It is believed to account for more than 12 million deaths annually [1]. Although it was previously demonstrated that dietary RPO protects the heart against ischemia/reperfusion-induced injury [2], the precise molecular mechanisms are still unclear.

RPO is a natural oil obtained from oil palm fruit (*Elaeis guineensis*). It is high in palmitic acid (44%) and oleic acid (40%) with natural fat-soluble tocopherol, tocotrienol and carotonoids, which may act as antioixants. Despite the high saturated fat content of RPO, several studies have demonstrated that RPO is associated with better recovery and protection of hearts submitted to ischaemia/reperfusion [2-4]. RPO-supplementation also caused differential phosphorylation of the MAPKs which were associated with improved functional recovery and reduced apoptosis [3,4]. This indicated that the improved physiological function associated with RPO-supplementation, was due to the cellular signaling effects of RPO, both through the NO-cGMP pathway or the pro-survival Akt pathway. These studies suggest that a combination of carotonoids, lycopene, pro-vitamin E and fatty acids provide more protections than one individual component [5-7].

The serine/threonine protein kinase, protein kinase B (a member of the PI3-K pathway), is a crucial regulator of widely divergent cellular processes including apoptosis (programmed cell death), cell proliferation, differentiation and metabolism [8,9]. Several researchers have reported that activation of PI3-K and Akt play an important role in promoting cardiomyocyte survival and function in models of cardiac injury [10,11]. Therefore, disruption of normal PI3-K signaling pathway during ischaemia/reperfusion-induced injury should therefore be considered as a potential target for therapy.

However, as far as we know, no evidence exits for the interaction between RPO and the PI3-K signaling pathway during reperfusion. In order to assess the possible mechanisms of protection, the isolated perfused rat heart model with the aid of a PI3-K inhibitor, wortmannin (Wn) was used to assess signaling proteins after RPO-supplementation.

## Results

### Percentage Rate Pressure Product Recovery (% RPP) (Figure 1)

**Figure 1:**
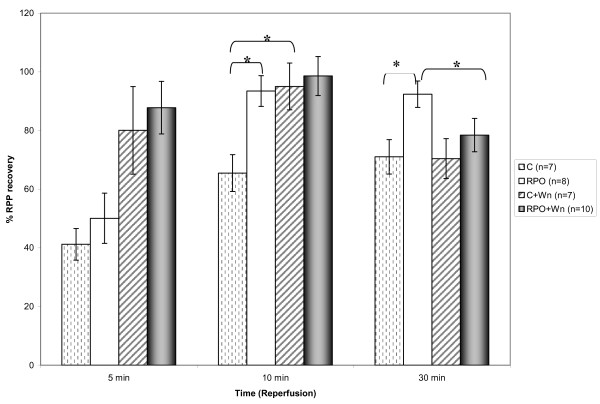
**Rate Pressure Product Recovery (%)**. The effect of RPO-supplementation and Wortmannin administration on RPP recovery during reperfusion (n = 7–10 per group) (mean +/- SEM), (* p < 0.05 for indicated groups).

RPO-supplementation caused an increase in % RPP recovery at 10 min during reperfusion when compared with the control group (65.5 ± 6.3% in control *vs *93.5 ± 5.2% in RPO; p < 0.05) confirming results in previous similar studies. Our results also indicate that C+Wn had an increased % RPP at the same time point, when compared to the control group (65.5 ± 6.3% in control *vs *95.0 ± 8.0% in C+Wn; p < 0.05). After 30 minutes of reperfusion, the % RPP in the RPO group was still significant higher compared to the control group (71.03 ± 5.82% in control *vs *92.36 ± 4.48% in RPO; p < 0.05). However, at this time point, there was also a significant difference between the RPO and the RPO+Wn groups (92.36 ± 4.48% in RPO *vs *75.21 ± 5.23% in RPO+Wn; p < 0.05). Table 1 shows the pre-ischaemic values of heart rate, developed pressure, rate pressure product and coronary flow in control and red palm oil groups. These values were used to calculate the % RPP.

**Table 1:** Pre-ischaemic values of heart rate, developed pressure, rate pressure product and coronary flow in control and red palm oil groups

	Heart Rate (beats/min	LVDev P (mmHg)	Coronary Flow (ml/min)	Rate Pressure Product (HR X LVDevP)
Control	296.6 ± 13.5	92.4 ± 3.9	16.7 ± 1.8	26314 ± 1973
Control + W	290.0 ± 10.8	75.1 ± 5.7^&^	12.5 ± 2.3	21778 ± 1940
RPO	312.0 ± 10.7	75.7 ± 3.5^$^	17.2 ± 1.0	23630 ± 1330
RPO + W	276.2 ± 13.9	67.2 ± 2.4^#^	12.2 ± 1.5	19104 ± 1389*

### The effect of RPO and wortmannin (Wn) on the regulatory subunit (p85) of PI3-Kinase (Figure 2)

**Figure 2:**
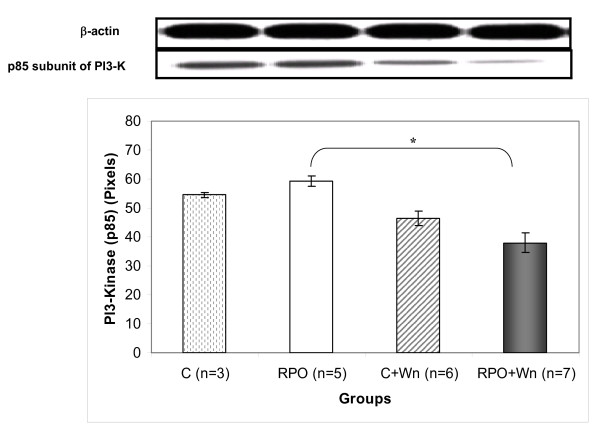
The effect of RPO-supplementation and Wortmannin administration on PI3-K (p85) activity in hearts subjected to ischaemia/reperfusion (n = 3–7 per group) (mean +/- SEM), (* p < 0.05 for indicated groups).

RPO+Wn significantly decreased PI3-K (p85) activity compared to RPO alone (37.9 ± 3.4 pixels *vs *59.2 ± 1.8 pixels; p < 0.05).

### The effect of RPO and wortmannin (Wn) on PKB/Akt (Ser^473^) phosphorylation (Figure 3)

**Figure 3:**
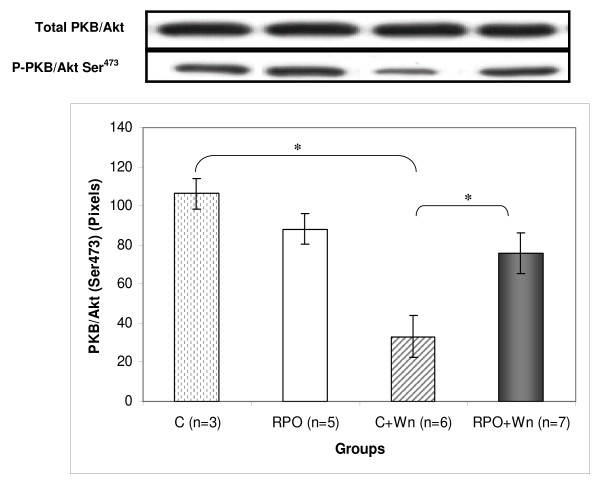
The effect of RPO-supplementation and Wortmannin administration on PKB/Akt (Ser473) phosphorylation in hearts subjected to ischaemia/reperfusion (n = 3–7 per group) (mean ± SEM) (* p < 0.05 for indicated groups).

Wortmannin administration during perfusion caused a significant decrease in PKB/Akt (Ser^473^) phosphorylation compared to the control group (33.2 ± 10.7 pixels for C+Wn *vs *106.4 ± 7.8 pixels for C; p < 0.05). RPO counteracted this decrease in phosphorylation when the RPO-fed animals received wortmannin during perfusion (33.2 ± 10.7 pixels in C+Wn *vs *75.87 ± 10.3 pixels in RPO+Wn; p < 0.05).

### The effect of RPO and wortmannin (Wn) on FKHR (Ser^256^) phosphorylation (Figure 4)

**Figure 4:**
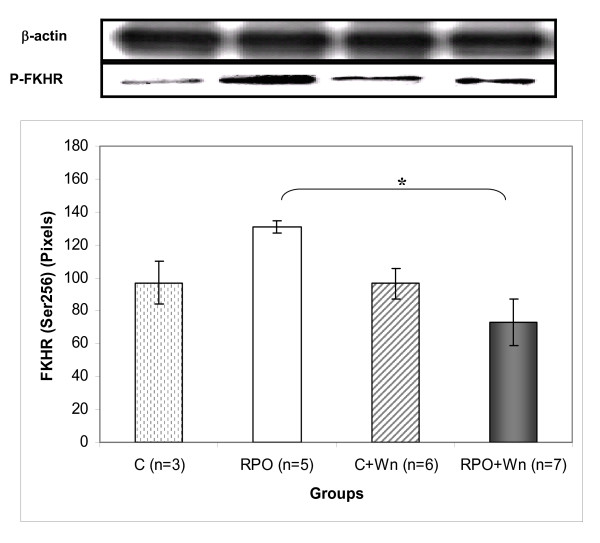
The effect exerted by RPO-supplementation and Wortmannin administration on FKHR phosphorylation in hearts subjected to ischaemia/reperfusion (n = 3–7 per group) (mean ± SEM) (* p < 0.05 for indicated groups).

There was a significant decrease in FKHR phosphorylation in the RPO+Wn group when compared with the RPO group (130.9 ± 3.5 pixels *vs *73.00 ± 14.1 pixels; p < 0.05).

### The effect of RPO and wortmannin (Wn) on Caspase-3 cleavage (Figure 5)

**Figure 5:**
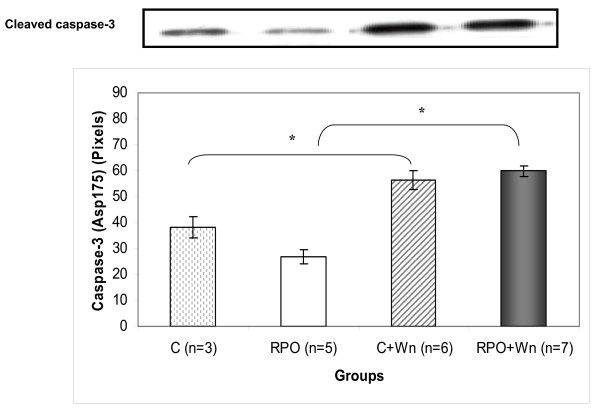
The effect exerted by RPO-supplementation and Wn administration on the cleavage of caspase-3 in hearts subjected to ischaemia/reperfusion (n = 3–7 per group) (mean ± SEM) (* p < 0.05 for indicated groups).

Wortmannin significantly increased caspase-3 cleavage compared with the control group (38.21 ± 4.14 pixels *vs *56.27 ± 3.63 pixels; p < 0.05). Interestingly, wortmannin also caused a significant increase in caspase-3 cleavage in the RPO group compared with the RPO control group (26.75 ± 2.6 pixels *vs *59.79 ± 2.1 pixels; p < 0.05).

### The effect of RPO and wortmannin (Wn) on PARP cleavage (Figure 6)

**Figure 6:**
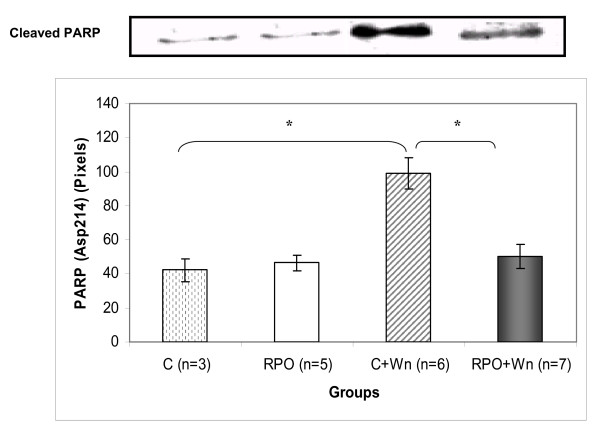
The cleavage of PARP in hearts subjected to ischaemia/reperfusion that received RPO-supplementation and Wortmannin administration (mean ± SEM) (n = 3–7 per group) (* p < 0.05 for indicated groups).

There was an increase in cleaved PARP in the control+Wn group when compared to the control group (42.12 ± 7.0 pixels *vs *98.9 ± 9.3 pixels; p < 0.05. PARP cleavage was significantly reduced in the RPO+Wn group compared with the control wortmannin group (98.9 ± 9.3 *vs *50.23 ± 6.9 pixels; p < 0.05.

## Discussion

We have demonstrated that dietary RPO supplementation offered significant protection against ischemia/reperfusion-induced injury in the isolated perfused working heart as reflected by improving functional recovery after 10 and 30 minutes of reperfusion. Furthermore, we have demonstrated for the first time that this effect of RPO was significantly attenuated when these hearts were perfused with the PI3-Kinase inhibitor, wortmannin. Therefore, we can conclude that the RPO-induced protection of the heart during ischemia/reperfusion-induced injury is, at least in part, mediated by the PI3-kinase pathway.

In response to ischemia/reperfusion-induced injury, cells activate various signal transduction pathways which either may be harmful or allow adaptation to this stressful environment. There is an increasing body of evidence showing that PKB/Akt may represent a nodal point to coordinate growth factor signaling in the early phase of ischemia/reperfusion-induced injury in the heart [12,13]. However, no knowledge exists about the possible involvement of the PI3-K/Akt pathway in RPO-induced protection during ischemia/reperfusion injury in the heart. Therefore, another aim of this study was to determine whether PI3-Kinase inhibition induced changes in protein phosphorylation and apoptosis in hearts subjected to ischemia/reperfusion injury of animals which received dietary RPO supplementation.

Upon growth factor activation of receptor tyrosine kinases, PI3-K is recruited to the receptor in the plasma membrane and phosphorylates phosphatidylinositol-4,5-bisphosphate (PIP_2_) on the 3-OH group, generating phosphatidylinositol-3,4,5-trisphosphate (PIP_3_). PI3-kinase is considered one of the intracellular signals responsible for the transmission of anti-apoptotic signals for controlling cell survival. Over expression of PI3-kinase in cells has been shown to cause a significant increase in survival of cells exposed to ionizing radiation [14]. Kennedy and co-workers also reported that inhibition of PI3-K causes an increase in apoptosis and a decrease in cell survival [15]. PI3-kinases are composed of a catalytic subunit (p110) and a regulatory subunit (p85). PI3-kinase inhibition with wortmannin significantly reduced the regulatory subunit (p85) when compared with the RPO control group. This decrease in PI3-K activity also correlates with reduced function after 30 minutes of perfusion in the isolated hearts in the RPO+Wn group compared to the RPO control group. Results in previous studies (2, 3, 4) showed that the signaling pathways were activated early in reperfusion whilst the true functional effect of these biochemical changes were only observed at later time points.

PKB/Akt is one of the most important targets of PI3-K because it phosphorylates and regulates a wide variety of proteins implicated in cell survival/death decisions. Activation of PKB requires binding to PIP_3 _via the pleckstrin homology domain and phosphorylation of Thr^308 ^in the activation loop as well as phosphorylation of Ser^473 ^within the carboxy-terminal [16]. The present results reveal that wormannin significantly reduced PKB (Ser^473^) phosphorylation when compared to the control group and that this reduction was partly counteracted in the RPO+Wn group. It was previously demonstrated (3) that PKB could play a role in RPO protection. In the current study the protective effect of RPO was abolished in the RPO+Wn group at 30 minutes of reperfusion. This indicates that PI3-K pathway may have had an effect on the RPO-induced protection. Although there was no significant decrease in PI3-K in the RPO group, PI-3K was significantly reduced in the RPO+Wn group.

Although PKB promotes cell survival, the mechanisms involved have only recently begun to emerge. One means by which PKB may promote cell survival is by directly phosphorylating transcription factors that control the expression of pro- and anti-apoptotic genes. PKB appears to both negatively regulate factors that promote the expression of death genes and positively regulate factors that induce survival genes [17,18]. An example is the family of forkhead transcription factors (FKHR). All the members of the FKHR family contain a PKB phosphorylation sequence which can be effectively phosphorylated by PKB *in vitro *[19,20]. Phosphorylation of FKHR by PKB alters its subcellular location. FKHR phosphorylation was significantly inhibited when RPO+Wn was compared to the RPO group. This leads to forkhead proteins residing predominantly in the nucleus where they are able to promote transcription of pro-apoptotic genes such as *Fas-L *and *Bim *through specific DNA elements in their promotor regions [21,22]. This result also correlates with the attenuation in function recovery in the RPO+Wn group compared to the control RPO group. Phosphorylation of FKHR by PKB leads to the export of FKHR from the nucleus and its accumulation and sequestration by 14-3-3 proteins in the cytoplasm [22]; thus inhibiting apoptosis.

Another hallmark of the apoptotic pathway is the cleavage of caspase-3. Wortmannin caused significant increases in caspase-3 cleavage in the control and in the RPO group, thereby promoting apoptosis in these groups. Furthermore, wortmannin also induced a significant increase in PARP cleavage to its proteolyzed products, a phenomenon that is well known to result from caspase-3 activation [19,20]. Interestingly, this increase cleavage of PARP was not observed in the RPO+Wn group. However, it is possible that an increase in this group may be seen if the reperfusion period is extended.

Currently there is no clear evidence that a single substance in the red palm oil is responsible for the protection or effect on the signaling pathways. Previous studies suggest that a combination of carotonoids and vitamin E in the presence of lycopene in a natural food supplement have a far more potent anti-oxidative effect [5-7] than when consumed in an isolated form.

## Conclusion

The present study demonstrates that the beneficial effect of RPO during ischemia/reperfusion-induced injury is partially mediated by the PI3-kinase signaling pathway. PI3-K inhibition attenuated functional recovery of the hearts during reperfusion. This attenuation in functional recovery when PI3-K was inhibited also correlated with reduced PKB and FKHR phosphorylation. This, in turn, leads to increased apoptosis as indicated by increased caspase-3 and PARP cleavage. The beneficial effect of RPO during ischemia/reperfusion-induced injury is thus associated with the PI3-K/PKB signaling pathway and thus points towards this pathway as a potential therapeutic target. The effects of RPO on cardiac function should be further characterized for the purpose of development as an agent for the management of ischemic injury.

## Methods

All animals received humane care in accordance with the *Principles of Laboratory Animal Care of the National Society of Medical Research *and the *Guide for the Care and use of Laboratory Animals of the National Academy of Sciences *(National Institutes of Health publications no. 80-23, revised 1978).

### Experimental Groups

Male Wistar groups were randomly divided into four groups: two control groups receiving standard rat chow and two experimental groups receiving standard rat chow plus 2 ml RPO (Carotina Premium) for 4 weeks. The composition of Carotino Premium red palm oil (per 100 ml) is given in Table 2. Red palm oil was mixed with one pellet of the chow every morning. Rats were only fed the rest of the daily rat chow allowance after they consumed the pellet with the red palm oil.

**Table 2:** The components of Carotino Premium red palm oil (per 100 ml)

Total fats	92 g
Monounsaturates	43 g
Polyunsaturates	12 g
Saturates	37 g
Trans fat	0 g
Cholesterol, Sodium	0 g
Protein, Carbohydrate, Dietary fibre	0 g
Natural Carotenes	46 mg
Natural Vitamin E	74 mg
Co-Enzyme Q10	4 mg

LVDevP = Left ventricular developed pressure; RPO = red palm oil; W = wortmanin

P < 0.05* RPO+W vs Control

^# ^RPO+W vs Control

^$^RPO vs Control

^&^Control + W vs Control

### Heart Perfusion

Rats weighing 300–400 g were anaesthetized with sodium pentobarbital, before hearts were rapidly excised and briefly rinsed by immersion in ice-cold Krebs-Henseleit buffer. Hearts were transferred to a Langendorff perfusion apparatus and perfused with a Krebs-Henseleit buffer equilibrated with 95% O_2 _and 5% CO_2 _at 37°C (118, 5 mM NaCl; 4.75 mM KCL; 1.2 mM MgCl • 6 H_2_O; 1.36 mM CaCl_2_; 25, 0 mM NaHCO_3_; 1.2 mM KH_2_PO_4_; 11, 0 mM glucose). Pressure was kept constant at 100 cm H_2_O. The aorta was cannulated and retrograde perfusion was initiated. Hearts were kept in a water-jacketed chamber to maintain temperature at 37°C. Immediately after cannulation, excess tissue and the left atrium was removed. A water-filled balloon (made from transparent sandwich wrap), connected to a pressure transducer, was inserted through the opening of the left atrium into the left ventricle. The pressure transducer was connected to a Powerlab system (ADInstruments Pty Ltd. Castle Hill, Australia) on a computer. After insertion, the balloon was inflated to 2 mmHg, and the contraction force of the heart against the balloon causes pressure on the fluid filled balloon. This pressure is then registered on the Powerlab system. Thus, systolic pressure, diastolic pressure and heart rate were measured. The first 10 min of perfusion was used to stabilize the heart.

### Perfusion Protocol

The study was divided into two perfusion protocols. In the first protocol, hearts were perfused for 10 min stabilization, followed by 20 min, during which mechanical function was documented. Hearts were then subjected to 25 min of total gloabal ischaemia. After the ischaemic period, hearts were reperfused for 30 min and mechanical function was again documented. To reduce the incidence of arrhythmias during reperfusion, a 2% lignocaine solution was used for the last min of pre-ischaemia perfusion as well as the initial 3 min of reperfusion in all hearts. In the second protocol, hearts were stabilized for 10 min and perfused for 15 min, before being subjected to a wortmannin solution (100 nM) for 5 min pre-ischaemia. After the 25 min total global ischaemic period, hearts were reperfused for 3 min with the wortmannin solution, before reverting to the drug-free Krebs-Henseleit buffer for the rest of the 27 min reperfusion period. Functional and biochemical measurements were taken.

### Mechanical Function Parameters measured

Functional measurements were taken during pre-ischaemia (20 min perfusion) and at 5 min, 10 min and 30 min into reperfusion. Heart rate and left ventricle developed pressure (LVDevP) were measured. LVDevP was calculated as the difference between left ventricular systolic (LVSP) and diastolic (LVDP) pressures. The rate pressure product (RPP) was calculated as the product of heart rate and LVDevP.

### Biochemical Analysis

To assess myocardial biochemical function, hearts, from all groups, were freeze clamped 10 min into reperfusion with Wollenberger clamps precooled in liquid nitrogen. Cardiac proteins were extracted with a lysis buffer containing: 20 mM Tris; 20 mM p-nitrophenylphosphate; 1 mM EGTA; 50 mM NaF; 0.1 sodium orthovanadate; 1 mM phenylmethyl sulfonyl fluoride (PMSF); 1 mM dithiothreitol (DTT); 10 μg/ml aprotinin. The tissue lysates were diluted in Laemmli sample buffer, boiled for 5 min and 60 μg protein was separated by 10% PAGE-SDS gel electrophoresis. The lysate protein content was determined using the Bradford technique [23]. The separated proteins were transferred to a PVDF membrane (Immobilon P, Millipore). These membranes were routinely stained with Ponceau Red for visualization of proteins. Nonspesific binding sites on the membranses were blocked with 5% fat-free milk in Tris-buffered saline – 0.1% Tween 20 (TBST) and then incubated with the primary antibodies that recognize PKB/Akt (Ser^473 ^and Thr^308^) and total PKB/Akt, PI3-K (p85), PDK1 (Ser^241^), FKHR (Ser^256^), GSK-3β (Ser^9^), cleaved caspase-3 (Asp^175^), cleaved PARP (Asp^214^) and PTEN (Ser^380^). Membranes were subsequently washed with large volumes of TBST (5 × 5 min) and the immobilized antibody conjugated with a diluted horseradish peroxidase-labaled secondary antibody (Amersham, LIFE SCIENCE). After thorough washing with TBST, membranes were covered with ECL detection reagents and quickly exposed to an autoradiography film (Hyperfilm ECL, RPN 2103) to detect light emission through a non-radioactive method (ECL Western blotting). Films were densitometrically analyzed (UN-SCAN-IT, Silkscience) and phoshorylated protein values were corrected for minor differences in protein loading, if required. Antibodies were purchased from Cell Signalling Technology and all other chemicals were obtained from Sigma (St Louis, Missouri, USA).

### Data analysis

Values are expressed as mean ± standard error of the mean (SEM). Some functional values are presented as percentage change from the baseline values. Results were compared by using a one-way ANOVA with a Bonferoni Multiple Comparison as a post hoc test. P < 0.05 was considered as statistically significant.

## Abbreviations

A: beta; C: control; cm: centimeter; CO_2_: carbon dioxide; DTT: Dithiothreitol; FKHR: Forkhead transcription factor; g: gram; LVDevP: Left ventricular developed pressure; Min: minutes; ml: milliliter; mM: millimolar; O_2_: oxygen; %: percentage; PMSF: Phenylmethyl sulfonyl fluoride; PTEN: Phoshoinositide-lipid-3-phosphotase; PIP_3_: Phosphatidylinositol-3,4,5-trisphosphate; PDK-1: Phosphoinositide-dependent kinase-1; PI-3K: Phosphatidylinositol 3-kinase; PARP: Poly(ADP-ribose) polymerase RPP: rate pressure product; ROS: reactive oxygen species; RPO: red palm oil; PKB/Akt: Serine/threonine protein kinase, protein kinase B or AKT; SEM: Standard error of the mean; H_2_O: water; Wn: wortmannin.

## Competing interests

The authors declare that they have no competing interests.

## Authors' contributions

AME contributed to the interpretation of results, drafted and finalized the manuscript. LO fed the rats, performed perfusions and western blots, initial preparation of manuscript. EFDT participated in design and interpretation of results. KK performed initial western blots. TC coordinated biochemical analysis and study design. PF contributed to the interpretation of the results concerning inhibitors and draft of manuscript. JvR conceived the study, participated in design, coordination, and interpretation of results and final preparation of manuscript.
